# Mixed stones: urinary stone composition, frequency and distribution by gender and age

**DOI:** 10.1007/s00240-023-01521-8

**Published:** 2024-01-08

**Authors:** Roswitha Siener, Jakob Rüdy, Helena Herwig, Marie-Therese Schmitz, Reinhold M. Schaefer, Philipp Lossin, Albrecht Hesse

**Affiliations:** 1 Department of Urology, University Stone Center, University Hospital Bonn, Venusberg-Campus 1, 53127 Bonn, Germany; 2 Department of Medical Biometry, Informatics and Epidemiology, Medical Faculty, Bonn, Germany; 3 Urinary Stone Analysis Center Bonn, Bonn, Germany

**Keywords:** Stone composition, Urolithiasis, Kidney stones, Calcium oxalate, Carbonate apatite, Sex

## Abstract

Proper analysis of urinary stone composition is a cornerstone for diagnosis, targeted treatment and recurrence prevention of urolithiasis. The aim of this study was to determine the composition, frequency and distribution of mixed stones according to gender and age of patients. A total of 42,519 urinary stones from 30,311 men and 12,208 women submitted between January 2007 and December 2020 were studied. Most urinary calculi consisted of two components (50.9%), followed by stones of a single constituent (27.1%) and three-component stones (21.9%), while four-component stones were only rarely identified (0.1%). Among all stones, 49.8% consisted of whewellite (COM), weddellite (COD), and mixtures of COM and COD, 33.8% were pure carbonate apatite (CA) and mixtures of CA with COM and/or COD, while 7.6% were composed of uric acid anhydrous (UAA), uric acid dihydrate (UAD), and mixed UAA and UAD. The remaining 8.8% of calculi were rare single-component stones and rare mixtures of various constituents. The number of stone components was inversely associated with age (p < 0.001). The proportion of men decreased significantly with the number of stone constituents, from 3.01:1 for single-component stones to 1.0:1 for four-component urinary calculi (p < 0.001). The vast majority of urinary calculi consisted of two or more components in varying proportions. While age was inversely associated with the number of stone constituents, the proportion of women increased significantly from single-component to four-component urinary calculi. A significant proportion of mixed stones could present a challenge for diagnosis and targeted recurrence prevention.

**Supplementary Information:**

The online version contains supplementary material available at 10.1007/s00240-023-01521-8.

## Introduction

Urolithiasis is a highly prevalent disease worldwide that poses a significant economic burden on healthcare systems [1–3]. The cumulative recurrence rate of urinary stones has been reported to be about 50% at 10 years [4]. The consequences of a high recurrence rate include a deterioration in the quality of life of stone formers [5]. Nephrolithiasis may also increase the risk of developing chronic kidney disease through several putative mechanisms, which differ according to the composition and size of stones [6]. Proper stone analysis is the essential prerequisite for the classification of the patient into risk groups, further diagnostic procedures, effective therapy and recurrence prevention of urinary stone formation [7–9].

Most previous studies on urinary stone composition have focused on the major stone component, which simplifies data analysis [10–14]. However, the majority of urinary calculi are mixed stones consisting of two or more components [15]. Knowledge of the different combinations and proportions of urinary stone constituents in specific mixtures as a function of gender and age might provide insight into the pathophysiological processes of urinary stone formation. Moreover, it is important to identify and quantify the individual components of each stone to ensure proper treatment of stone patients. However, findings of previous studies on the composition of mixed stones were based on a rather small number of cases [15–17].

In a study on a limited number of different mixtures of stone components, the influence of patient age was not considered [18]. Although data on the association between stone composition and age are available from a high-volume urinary stone analysis laboratory, stone composition has been determined using different methods [19]. The aim of this study was to investigate the exact composition and frequency of mixed stones and to determine the distribution by gender and age of patients in a large series of urinary stone analyses to gain insight into the characteristics and potential pathophysiological processes involved in the formation of the specific combinations.

## Materials and methods

### Stone analysis

A total of 42,519 urinary calculi were evaluated that were submitted to the Urinary Stone Analysis Center Bonn and the University Stone Center of the Department of Urology at Bonn University Hospital for analysis between 2007 and 2020. Urinary stone samples were collected from all over Germany. Calculi were obtained after spontaneous passage, chemolysis, lithotripsy, surgery or instrumental procedures. Only the first stone received per patient was included in this analysis. Patients with incomplete gender and age information were excluded from the study.

Each calculus was analyzed according to a standard operating procedure. The stones were dried at 37 °C and then ground into a fine, homogenized powder using an agate mortar. The analysis was carried out by Fourier-transformed infrared (FTIR) spectroscopy using the attenuated total reflectance (ATR) technique (PerkinElmer, Waltham, MA, USA). The resulting infrared spectrum was evaluated with a computerized library of reference spectra of all known single stone constituents and mixtures [20], and double-checked by qualified and trained personnel to ensure accurate analysis. Laboratory quality certification was available for the stone analysis. The FTIR spectroscopy method is considered the gold standard for routine clinical analysis of stone composition [12].

### Statistical analysis

Descriptive statistics were calculated regarding the frequency of each stone type, age, and gender of patients. Statistical comparison of the age between men and women was performed with the Mann Whitney U-test. The association between gender and the type of stone and between gender and the number of stone components was assessed using the chi-square test. Fisher’s exact test was used when the chi-square test was not applicable. Correlations between variables were calculated using Spearman’s rank correlation. The significance level was set at 0.05 and p-values < 0.05 were considered statistically significant. As the study was exploratory in nature, adjustments for multiple testing were not performed. Statistical analyses were carried out using SPSS for Windows, version 28.

## Results

### Stone composition

Of the 42,519 urinary stones included in the evaluation, 50.9% were composed of two components, followed by single-component stones (27.1%), and calculi consisting of three components (21.9%), while four-component stones were only rarely encountered (0.1%) (Tables 1, 2, 3, Supplementary Table 1). In all, 17 major components and 75 combinations of stone constituents were identified.

**Table 1:** Distribution of single-component stones

Stone component	Total			Men			Women			P *	P **	M/F	rM/F
	Number		Age	Number		Age	Number		Age				
	n	%	M ± SD	n	%	M ± SD	n	%	M ± SD				
Calcium oxalates													
COM	6709	58.1	55.5 ± 14.4	5186	59.8	55.7 ± 14.0	1523	53.0	54.8 ± 15.4	< 0.001	0.226	3.41	1.13
COD	676	5.9	45.5 ± 15.4	523	6.0	44.3 ± 14.7	153	5.3	49.8 ± 16.7	0.159	< 0.001	3.42	1.13
Uric acid and urates													
UAA	2352	20.4	64.0 ± 12.7	1932	22.3	63.9 ± 12.6	420	14.6	64.4 ± 13.4	< 0.001	0.408	4.60	1.53
UAD	122	1.1	57.9 ± 12.3	102	1.2	58.6 ± 12.6	20	0.7	54.5 ± 10.5	0.029	0.213	5.10	1.69
Ammonium urate	69	0.6	47.5 ± 18.8	42	0.5	51.2 ± 19.2	27	0.9	41.6 ± 16.8	0.006	0.036	1.56	0.52
Sodium/potassium urate	36	0.3	57.8 ± 18.7	26	0.3	57.5 ± 20.2	10	0.3	58.6 ± 14.7	0.690	0.986	2.60	0.86
Phosphates													
CA	761	6.6	51.1 ± 18.8	327	3.8	55.4 ± 18.4	434	15.1	47.9 ± 18.6	< 0.001	< 0.001	0.75	0.25
Brushite	209	1.8	44.3 ± 17.2	160	1.8	44.3 ± 17.5	49	1.7	44.2 ± 16.3	0.622	1.000	3.27	1.08
Struvite	74	0.6	65.7 ± 19.7	35	0.4	68.4 ± 19.4	39	1.4	63.3 ± 19.9	< 0.001	0.442	0.90	0.30
Other phosphates	36	0.3	50.8 ± 19.0	17	0.2	55.3 ± 14.4	19	0.7	46.8 ± 21.9	0.001	0.325	0.89	0.30
Protein													
Protein	170	1.5	59.8 ± 16.4	119	1.4	60.7 ± 15.9	51	1.8	57.6 ± 17.4	0.122	0.337	2.33	0.77
Genetically determined stones													
Cystine	184	1.6	41.3 ± 20.3	118	1.4	40.7 ± 19.2	66	2.3	42.2 ± 22.1	< 0.001	0.896	1.79	0.59
2,8-Dihydroxyadenine	4	< 0.1	27.5 ± 18.8	2	< 0.1	20.5 ± 29.0	2	0.1	34.5 ± 5.0	0.260	1.000	1.00	0.33
Xanthine	0	–	–	0	–	–	0	–	–	–	–	–	–
Others													
Artifacts	46	0.4	52.9 ± 17.5	28	0.3	54.7 ± 17.1	18	0.6	50.1 ± 18.2	0.025	0.506	1.56	0.52
Silicate	55	0.5	50.6 ± 18.4	29	0.3	47.0 ± 17.1	26	0.9	54.6 ± 19.4	< 0.001	0.056	1.12	0.37
Calcite	21	0.2	50.5 ± 21.0	10	0.1	53.0 ± 19.6	11	0.4	48.3 ± 23.0	0.004	0.349	0.91	0.30
Drugs	15	0.1	55.7 ± 11.3	9	0.1	52.6 ± 11.4	6	0.2	60.3 ± 10.4	0.176	0.215	1.50	0.50
Total	11,539	100	56.0 ± 15.7	8,665	100	56.5 ± 15.2	2,874	100	54.4 ± 17.5		< 0.001	3.01	1.00

* P value for comparison between genders; ** P value for comparison of age; M/F male-to-female ratio; rM/F relative male-to-female ratio

**Table 2:** Distribution of two-component stones

Stone component	Total			Men			Women			P *	P **	M/F	rM/F
	Number		Age	Number		Age	Number		Age				
	n	%	M ± SD	n	%	M ± SD	n	%	M ± SD				
Calcium oxalates													
COM													
COM-COD	13,794	63.8	51.5 ± 14.3	10,692	69.7	51.6 ± 13.9	3102	49.3	51.3 ± 15.6	< 0.001	0.792	3.45	1.41
COM-CA	1216	5.6	50.0 ± 15.3	691	4.5	50.7 ± 15.4	525	8.4	49.1 ± 15.2	< 0.001	0.103	1.32	0.54
COM-UAA	886	4.1	61.4 ± 13.5	698	4.5	61.3 ± 13.3	188	3.0	62.0 ± 14.3	< 0.001	0.352	3.71	1.52
COM-UAD	18	0.1	57.9 ± 10.9	17	0.1	57.1 ± 10.6	1	< 0.1	72.0	0.034	0.148	17.00	6.97
COM-protein	8	< 0.1	64.8 ± 17.3	6	< 0.1	61.7 ± 19.0	2	< 0.1	74.0 ± 8.5	1.000	0.317	3.00	1.23
COM-struvite	4	< 0.1	58.8 ± 13.2	1	< 0.1	40.0	3	< 0.1	65.0 ± 5.2	0.077	0.500	0.33	0.14
COM-brushite	3	< 0.1	40.3 ± 12.3	3	< 0.1	40.3 ± 12.3	0	–	–	0.561	–	–	–
COM-ammonium urate	3	< 0.1	66.0 ± 21.9	2	< 0.1	61.5 ± 29.0	1	< 0.1	75.0	1.000	1.000	2.00	0.82
COM-drugs	2	< 0.1	55.0 ± 11.3	1	< 0.1	47.0	1	< 0.1	63.0	0.497	–	1.00	0.41
COM-phosphates	1	< 0.1	70.0	1	< 0.1	70.0	0	–	–	1.000	–	–	–
COD													
COD-CA	3381	15.6	44.0 ± 16.3	1854	12.1	43.8 ± 16.5	1527	24.3	44.3 ± 16.1	< 0.001	0.322	1.21	0.50
COD-brushite	213	1.0	46.0 ± 17.0	161	1.0	44.4 ± 17.0	52	0.8	50.8 ± 16.1	0.133	0.013	3.10	1.27
COD-protein	10	< 0.1	58.3 ± 18.9	4	< 0.1	70.0 ± 6.5	6	0.1	50.5 ± 20.8	0.041	0.067	0.67	0.27
COD-phosphates	9	< 0.1	42.9 ± 14.4	4	< 0.1	46.3 ± 18.0	5	0.1	40.2 ± 12.4	0.133	0.325	0.80	0.33
COD-struvite	8	< 0.1	50.9 ± 19.8	5	< 0.1	47.6 ± 25.3	3	< 0.1	56.3 ± 4.5	0.699	0.180	1.67	0.68
COD-UAA	7	< 0.1	60.0 ± 9.6	5	< 0.1	61.2 ± 7.8	2	< 0.1	57.0 ± 17.0	1.000	0.857	2.50	1.02
COD-UAD	2	< 0.1	67.5 ± 13.4	2	< 0.1	67.5 ± 13.4	0	–	–	1.000	–	–	–
COD-ammonium urate	2	< 0.1	41.0 ± 12.7	2	< 0.1	41.0 ± 12.7	0	–	–	1.000	–	–	–
COD-silicate	1	< 0.1	52.0	0	–	–	1	< 0.1	52.0	0.291	–	–	–
Uric acid and urates													
UAA-UAD	746	3.4	60.9 ± 11.9	603	3.9	60.8 ± 11.8	143	2.3	61.0 ± 12.4	< 0.001	0.952	4.22	1.73
UAA-ammonium urate	3	< 0.1	54.3 ± 28.5	1	< 0.1	54.0	2	< 0.1	54.5 ± 40.3	0.204	1.000	0.50	0.20
UAA-brushite	3	< 0.1	58.7 ± 5.1	0	–	–	3	< 0.1	58.7 ± 5.1	0.025	–	–	–
UAA-protein	3	< 0.1	63.7 ± 19.3	2	< 0.1	60.5 ± 26.2	1	< 0.1	70.0	1.000	1.000	2.00	0.82
UAD-ammonium urate	3	< 0.1	49.3 ± 6.4	3	< 0.1	49.3 ± 6.4	0	–	–	0.561	–	–	–
UAA-CA	2	< 0.1	74.0 ± 9.9	1	< 0.1	67.0	1	< 0.1	81.0	0.497	–	1.00	0.41
UAA-struvite	2	< 0.1	67.5 ± 19.1	2	< 0.1	67.5 ± 19.1	0	–	–	1.000	–	–	–
UAA-cystine	1	< 0.1	68.0	1	< 0.1	68.0	0	–	–	1.000	–	–	–
UAD-brushite	1	< 0.1	76.0	1	< 0.1	76.0	0	–	–	1.000	–	–	–
Ammonium urate-sodium/potassium urate	5	< 0.1	48.6 ± 27.9	4	< 0.1	56.3 ± 25.5	1	< 0.1	18.0	1.000	0.400	4.00	1.64
Phosphates													
CA-struvite	899	4.2	60.1 ± 18.8	368	2.4	61.8 ± 19.4	531	8.4	58.9 ± 18.3	< 0.001	0.002	0.69	0.28
CA-protein	83	0.4	52.2 ± 19.6	44	0.3	52.9 ± 23.0	39	0.6	51.3 ± 15.1	< 0.001	0.309	1.13	0.46
CA-brushite	48	0.2	45.4 ± 20.7	32	0.2	44.3 ± 18.6	16	0.3	47.6 ± 24.7	0.514	0.759	2.00	0.82
CA-ammonium urate	6	< 0.1	54.7 ± 29.0	5	< 0.1	49.8 ± 29.5	1	< 0.1	79.0	0.679	0.143	5.00	2.05
CA-cystine	1	< 0.1	29.0	0	–	–	1	< 0.1	29.0	0.291	–	–	–
Phosphates-protein	184	0.9	54.3 ± 17.2	81	0.5	53.8 ± 16.6	103	1.6	54.7 ± 17.7	< 0.001	0.713	0.79	0.32
Phosphates-calcite	7	< 0.1	61.0 ± 9.4	5	< 0.1	58.6 ± 10.2	2	< 0.1	67.0 ± 4.2	1.000	0.439	2.50	1.02
Phosphates-drugs	3	< 0.1	49.3 ± 22.0	1	< 0.1	63.0	2	< 0.1	42.5 ± 26.2	0.204	0.669	0.50	0.20
Phosphates-brushite	1	< 0.1	45.0	0	–	–	1	< 0.1	45.0	0.291	–	–	–
Struvite-ammonium urate	48	0.2	59.5 ± 19.3	33	0.2	63.5 ± 18.6	15	0.2	50.9 ± 18.6	0.738	0.024	2.20	0.90
Struvite-protein	6	< 0.1	54.3 ± 28.9	4	< 0.1	41.8 ± 27.4	2	< 0.1	79.5 ± 5.0	1.000	0.133	2.00	0.82
Struvite-cystine	2	< 0.1	38.5 ± 20.5	1	< 0.1	24.0	1	< 0.1	53.0	0.497	–	1.00	0.41
Brushite-protein	1	< 0.1	58.0	1	< 0.1	58.0	0	–	–	1.000	–		–
Others													
Silicate-calcite	2	< 0.1	52.0 ± 1.4	0	–	–	2	< 0.1	52.0 ± 1.4	0.084	–	–	–
Total	21,628	100	51.3 ± 15.6	15,342	100	51.6 ± 15.1	6.286	100	50.7 ± 16.6		< 0.001	2.44	1.00

* P value for comparison between genders; ** P value for comparison of age; M/F male-to-female ratio; rM/F relative male-to-female ratio

**Table 3:** Distribution of three-component stones

Stone component	Total			Men			Women			P *	P **	M/F	rM/F
	Number		Age	Number		Age	Number		Age				
	n	%	M ± SD	n	%	M ± SD	n	%	M ± SD				
Calcium oxalates													
COM-COD-CA	9005	96.5	46.7 ± 14.6	6121	97.3	46.6 ± 14.2	2884	95.0	46.9 ± 15.5	< 0.001	0.410	2.12	1.02
COM-COD-UAA	24	0.3	54.2 ± 20.5	18	0.3	52.4 ± 22.2	6	0.2	59.5 ± 14.8	0.429	0.581	3.00	1.45
COM-COD-brushite	13	0.1	49.5 ± 13.4	8	0.1	50.1 ± 15.0	5	0.2	48.6 ± 11.8	0.768	0.883	1.60	0.77
COM-COD-protein	11	0.1	66.6 ± 16.4	11	0.2	66.6 ± 16.4	0	–	–	0.021	–	–	–
COM-COD-struvite	3	< 0.1	47.0 ± 10.0	2	< 0.1	42.0 ± 7.1	1	< 0.1	57.0	1.000	0.667	2.00	0.97
COM-COD-urate	1	< 0.1	54.0	0	–	–	1	< 0.1	54.0	0.325	–	–	–
COM-CA-protein	4	< 0.1	70.5 ± 10.6	2	< 0.1	64.0 ± 8.5	2	0.1	77.0 ± 9.9	0.600	0.333	1.00	0.48
COM-UAA-CA	2	< 0.1	54.0 ± 8.5	1	< 0.1	48.0	1	< 0.1	60.0	0.545	–	1.00	0.48
COM-COD-phosphates	1	< 0.1	53.0	0	–	–	1	< 0.1	53.0	0.325	–	–	–
COM-UAA-ammonium urate	1	< 0.1	50.0	0	–	–	1	< 0.1	50.0	0.325	–	–	–
COD-CA-protein	17	0.2	50.5 ± 23.6	8	0.1	64.8 ± 17.8	9	0.3	37.9 ± 21.4	0.072	0.016	0.89	0.43
COD-protein-phosphates	3	< 0.1	51.3 ± 18.8	2	< 0.1	56.0 ± 24.0	1	< 0.1	42.0	1.000	1.000	2.00	0.97
COD-struvite-ammonium urate	1	< 0.1	45.0	1	< 0.1	45.0	0	–	–	1.000	–	–	–
COD-CA-UAA	1	< 0.1	30.0	1	< 0.1	30.0	0	–	–	1.000	–	–	–
COD-COM-drugs	1	< 0.1	38.0	0	–	–	1	< 0.1	38.0	0.325	–	–	–
Phosphates													
CA-COD-struvite	131	1.4	55.7 ± 18.2	53	0.8	59.7 ± 17.8	78	2.6	53.1 ± 18.1	< 0.001	0.042	0.68	0.33
CA-COD-brushite	39	0.4	43.6 ± 15.5	25	0.4	41.6 ± 14.8	14	0.5	47.1 ± 16.8	0.655	0.409	1.79	0.86
CA-COM-struvite	18	0.2	60.4 ± 23.5	8	0.1	64.8 ± 18.6	10	0.3	57.0 ± 27.3	0.037	0.573	0.80	0.39
CA-ammonium urate-struvite	12	0.1	60.6 ± 18.1	6	0.1	68.8 ± 6.7	6	0.2	52.3 ± 22.6	0.197	0.078	1.00	0.48
CA-brushite-struvite	3	< 0.1	56.0 ± 3.6	1	< 0.1	52.0	2	0.1	58.0 ± 1.4	0.249	0.221	0.50	0.24
CA-struvite-protein	3	< 0.1	76.7 ± 3.8	1	< 0.1	74.0	2	0.1	78.0 ± 4.2	0.249	0.221	0.50	0.24
CA-UAA-cystine	1	< 0.1	38.0	0	–	–	1	< 0.1	38.0	0.325	–	–	–
CA-UAA-protein	1	< 0.1	91.0	1	< 0.1	91.0	0	–	–	1.000	–	–	–
CA-struvite-UAA	1	< 0.1	58.0	0	–	–	1	< 0.1	58.0	0.325	–	–	–
CA-struvite-calcite	1	< 0.1	54.0	0	–	–	1	< 0.1	54.0	0.325	–	–	–
Phosphates-struvite-protein	1	< 0.1	10.0	1	< 0.1	10.0	0	–	–	1.000	–	–	–
Uric acid													
UAA-UAD-COM	28	0.3	58.6 ± 12.1	20	0.3	57.9 ± 12.0	8	0.3	60.3 ± 12.9	0.653	0.593	2.50	1.21
UAA-UAD-Protein	1	< 0.1	49.0	1	< 0.1	49.0	0	–	–	1.000	–	–	–
Total	9,328	100	47.0 ± 14.9	6,292	100	46.9 ± 14.4	3,036	100	47.2 ± 15.7		0.310	2.07	1.00

*P value for comparison between genders; ** P value for comparison of age; M/F male-to-female ratio; rM/F relative male-to-female ratio

Among the single-component stones, whewellite (calcium oxalate monohydrate; COM) was the most common component (58.1%) followed by uric acid anhydrous (UAA) (20.4%), carbonate apatite (CA) (6.6%) and weddellite (calcium oxalate dihydrate; COD) (5.9%) (Table 1). COM, UAA, and uric acid dihydrate (UAD) stones were obtained more frequently from men, whereas CA and struvite were significantly more common in women. The ratios of COM-to-COD and UAA-to-UAD were approximately 10:1 and 20:1, respectively, in both men and women.

The COM-COD combination was the most common two-component mixture (63.8%), followed by COD-CA (15.6%), COM-CA (5.6%), and CA-struvite (4.2%) (Table 2). The male-to-female ratio of COM-COD was 3.45, which is similar to that of the pure COM and COD stones, respectively, whereas the male-to-female ratio for mixed COM-CA, COD-CA, and CA-struvite was only 1.32, 1.21 and 0.69, respectively.

**Fig. 1:**
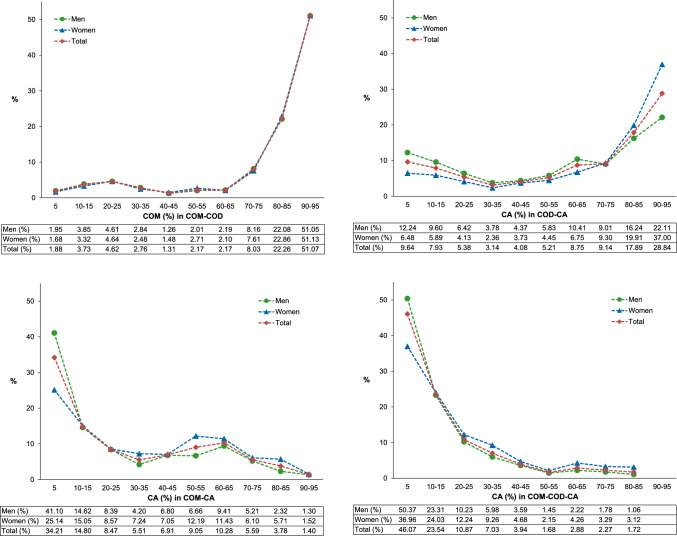
Proportion of stone component in mixed stones by gender a Proportion of COM (%) in COM-COD stones b Proportion of CA (%) in COD-CA stones c Proportion of CA (%) in COM-CA stones d Proportion of CA (%) in COM-COD-CA stones

Among the COM-COD stones, COM occurred predominantly with a proportion of more than 70% (Fig. 1a). The male-to-female ratio was similar at each proportion of COM in COM-COD stones. The COD-CA stones contained CA predominantly in proportions of 5% and 60% or more, respectively (Fig. 1b). The male-to-female ratio decreased from 2.29 at 5% CA to 0.73 at 90–95% CA. In contrast, CA was predominantly present in COM-CA stones in proportions below 30% (Fig. 1c).

By far the most common three-component mixture was COM-COD-CA, accounting for 96.5% of stones (Table 3). For the COM-COD-CA stones, the CA proportion was predominantly below 30% (Fig. 1d). The male-to-female ratio decreased from 2.89 at 5% CA to 0.72 at 80% or more CA. Moreover, four different four-component mixtures were identified, of which COM-COD-CA-struvite was the most common combination (Supplementary Table 1).

Among all calculi, calcium oxalates (CaOx) were the most common stone constituents (49.8%) and occurred as COM, COD, and mixed COM-COD. CA was the second most common stone component (36.8%). Of the 15,660 CA-containing calculi, 4.9% consisted of CA and 86.9% were mixed COM-CA (7.8%), COD-CA (21.6%), and COM-COD-CA (57.5%), accounting for 33.8% of all calculi. Mixed CA-struvite stones comprised 5.7% of the CA-containing calculi, while the remainder were mixtures of CA with various other stone components, such as CA-COD-struvite (0.8%), CA-protein (0.5%), and CA-brushite (0.3%). Among all stones, 7.6% were uric acid (UA) stones, i.e., consisted of UAA, UAD, and UAA-UAD. Among UA calculi, 76.4% were UAA, UAD, and UAA-UAD, while 22.9% were admixed with CaOx, i.e., COM and/or COD. In addition, mixtures of UAA and/or UAD were found with various other rather rare stone components, such as ammonium urate, brushite, protein, CA and struvite. The remaining 8.8% of all calculi were rare single-component stones and rare mixtures of various constituents.

The number of stone components was significantly inversely associated with age (R =  – 0.205; p < 0.001). The proportion of men decreased significantly with the number of stone constituents, from 3.01:1 for single-component stones to 2.44:1 for two-component, 2.07:1 for three-component, and 1.0:1 for four-component urinary calculi (p < 0.001).

## Discussion

Most previous studies on urinary stone composition have focused on the major stone component [10–14]. The few studies that evaluated the frequency of mixed stones were based on a rather small number of cases [15–17]. A study of 10,000 urinary calculi in patients from East Berlin, Germany, revealed that about 70% of stones were composed of more than one component [16], while in a study of 10,438 calculi conducted in France, 93.1% were classified as mixed stones [15]. According to a study in the Chinese population 66.8% of stones consisted of two or more constituents [18]. Although it is known that most stones contain more than one component, the exact composition and distribution by gender and age of the patients have not yet been analysed in large series.

In the present study of 42,519 urinary stones, 73% consisted of two or more components, accounting for the vast majority of all stones. CaOx, i.e., COM, COD, and mixed COM-COD, were the predominant stone types, comprising 49.8% of all calculi. The combination of COM-COD was the most frequent mixture, accounting for 32.4% of all stones, which is consistent with previous findings [16]. COM, the most common single-component stone, was substantially more abundant compared to COD, with a ratio of 10:1 in both genders. Moreover, in 85% of COM-COD stones, the proportion of COM was above 50%. Previous studies have already reported a higher proportion of COM compared to COD in both sexes [10, 14]. An explanation for the high proportion of COM compared to COD could be the formation process of the two crystal forms of CaOx. COM is the thermodynamically more stable hydrate form, while COD is metastable and is considered the primary phase of CaOx stone formation [21, 22]. The conversion of COD to COM in urinary calculi has been convincingly demonstrated [21–23]. The distinction between COM and COD and the COM-to-COD ratio is of clinical interest, especially when deciding on the stone removal method, since COM is difficult to disintegrate due to its density and hardness [24]. Knowing only the major constituent of a urinary stone may not allow adequate prediction of its fragility in lithotripsy treatment [25].

Although CA was the second most common stone constituent in the present study, accounting for 36.8% of all urinary stones, 95% were mixtures of CA with various stone components, including COM, COD, struvite, protein, brushite, and ammonium urate. The vast majority of CA-containing stones were combinations with COM and/or COD, accounting for 32.0% of all stones. In both COM-COD-CA and COM-CA stones, CA was mainly present in proportions below 30%, whereas the CA content in COD-CA stones was predominantly high. While the male-to-female ratio of COM, COD and COM-COD were similar at 3.41, 3.42 and 3.45, respectively, the male-to-female ratio for mixed COM-COD-CA, COM-CA and COD-CA were only 2.12, 1.32 and 1.21, respectively. Identification of CA and various other stone components in mixed stones is of interest in the etiology and treatment of urolithiasis. The predominantly low proportion of CA in COM-COD-CA and COM-CA stones suggests that growth over sites of (Randall’s) interstitial CA plaque appeared to be the mode of CaOx stone formation in these patients [26]. Because 50% of all urinary stones consisted of COM and/or COD without detectable CA, other mechanisms of CaOx stone formation are also conceivable. Another explanation could be that the percentage of CA in COM and/or COD stones was less than 5% and thus below the detection limit of the analytical method. Causes that contribute to the formation of CA-containing stones include conditions that result in a transient or persistently high urinary pH, hyperphosphaturia and hypercalciuria, such as primary hyperparathyroidism, complete and incomplete distal renal tubular acidosis, medullary sponge kidney, renal phosphate wasting disorders, abuse of absorbable antacids and drugs inducing proximal renal tubular acidosis (carbonic anhydrase inhibitors) [27, 28]. Although urinary tract infection is not a prerequisite for the formation of CA-containing stones, infectious conditions favour CA formation [27]. Treatment of mixed stones such as COM-CA, COD-CA, and COM-COD-CA can be challenging because therapy for CaOx stone disease includes urinary alkalization, whereas urinary acidification is indicated for CA stones [9, 29].

In the present study, UA stones, consisting of UAA, UAD, and mixed UAA-UAD, were the third most common type of stone, accounting for approximately 8% of all stones. Among the various constituents admixed with UAA and/or UAD, COM and/or COD were the most common components. These findings are consistent with previous studies [16, 17, 30]. The differentiation of UAA/UAD stones from non-UA calculi and the quantification of mixed components is critical because pure UA stones can be treated with oral chemolysis via urinary alkalization rather than surgical procedures [9, 31].

Rare single-component stones and rare mixtures of different constituents accounted for 8.8% of all stones. Rare stone types include, for example, cystine, brushite, struvite, 2,8-dihydroxyadenine, protein, ammonium, sodium and potassium urate, various mixtures of these stone constituents with each other and/or with COM, COD, UAA, UAD and CA. The majority of these stones are deemed at high risk of recurrence [9]. Rare mixtures of stone components may pose an additional challenge for diagnosis, treatment and recurrence prevention. Delay in recognizing and evaluating a rare stone disease in a patient may cause chronic kidney disease that would have been preventable [32].

Age was significantly inversely associated with the number of stone components. Furthermore, the proportion of men decreased significantly with the number of stone constituents. However, the reason for these associations remains unclear. Future research is needed to evaluate the impact of other factors on the formation of mixed stones, including comorbidities such as diabetes, hypertension and gout, and to assess the potential effects of medical management in changing stone composition trends. Even though information of how a stone might have been formed is lost when it is fragmented, such as the presence of Randall’s plaque, analysis of the fragments still allows for the mineral composition to be determined [8]. To clarify the causes and mechanisms of mixed stone formation, further studies on whole stones, i.e. spontaneously passed calculi and stones fully extracted by URS or PNL, are required. Although the observation of stone morphology is of potential use, more work needs to be done before it can be added to the standard stone analysis [28]. Since the current data confirm the frequency of mixed stones reported in previous studies, it can be assumed that the present findings can be generalized to countries other than Germany. This largest series of stone analysis to date differentiating between gender- and age-specific aspects should provide clues to the formation process of a number of different mixed stones.

## Conclusion

Urinary stones rarely consisted of a single component. The vast majority of urinary calculi contained two or more components in a wide variety of different combinations. While age was inversely related to the number of stone constituents, the proportion of women increased significantly from single-component to four-component urinary calculi. Mixed stones might present a challenge for evaluation, targeted therapy and recurrence prevention of urinary stone formation. Therefore, identification and quantification of the individual stone components in mixed stones is critical for the etiology, diagnosis, and personalized treatment of urolithiasis. Understanding the pathophysiologic processes involved in the formation of mixed stones is essential to ensure appropriate prevention of stone recurrences. A personalized approach that considers all clinically relevant stone constituents could improve the treatment outcome of urinary stone disease.

## Supplementary Information

Below is the link to the electronic supplementary material.

Supplementary file1 (DOCX 17 KB)

## Data Availability

The data presented in this study are available upon reasonable personal request.
