# Supplementary material for: Mixed stones: urinary stone composition, frequency and distribution by gender and age
Source: Urolithiasis. 2024 Jan 8;52(1):24. doi: 10.1007/s00240-023-01521-8 (PMC10774159; doi:10.1007/s00240-023-01521-8)
Supplement: Supplementary file 1 — Supplementary file1 (DOCX 17 KB) [file 240_2023_1521_MOESM1_ESM.docx]

**SupplementaryTable 1** Distribution of four-component stones

| **Stone component** | **Total** | | | **Men** | | | **Women** | | |  |  | **M/F** | **rM/F** |
| --- | --- | --- | --- | --- | --- | --- | --- | --- | --- | --- | --- | --- | --- |
|  | Number | | Age | Number | | Age | Number | | Age |  |  |  |  |
|  | n | % | M ± SD | n | % | M ± SD | n | % | M ± SD | P * | P ** |  |  |
| **Calcium oxalates** |  |  |  |  |  |  |  |  |  |  |  |  |  |
| COM-COD-CA-struvite | 14 | 58.3 | 45.6 ± 20.8 | 4 | 33.3 | 44.5 ± 19.8 | 10 | 83.3 | 46.1 ± 22.2 | 0.036 | 0.945 | 0.40 | 0.40 |
| COM-COD-CA-protein | 6 | 25.0 | 62.2 ± 8.8 | 5 | 41.7 | 62.4 ± 9.8 | 1 | 8.3 | 61.0 | 0.155 | 1.000 | 5.00 | 5.00 |
| COM-COD-CA-brushite | 1 | 4.2 | 61.0 | 1 | 8.3 | 61.0 | 0 | - | - | 1.000 | - | - | - |
| COM-COD-CA-UAA | 3 | 12.5 | 45.0 ± 8.0 | 2 | 16.7 | 45.0 ± 11.3 | 1 | 8.3 | 45.0 | 1.000 | 1.000 | 2.00 | 2.00 |
| **Total** | **24** | **100** | **50.3 ± 18.0** | **12** | **100** | **53.4 ± 15.4** | **12** | **100** | **47.3 ± 20.5** |  | **0.410** | **1.00** | **1.00** |

* P value for comparison between genders; ** P value for comparison of age; M/F male-to-female ratio; rM/F relative male-to-female-ratio
